# A Serious Question

**Published:** 1887-01-15

**Authors:** 


### A SERIOUS QUESTION.

"There are now two readings of the parable of the good Samaritan. Our modern theorists tell us that when the proud Levite and the bloated priest had passed by, a benevolent Communist came down that way and began to whisper in the ear of the wounded man: 'My friend, you have been much in error. You were a thief yourself when you accumulated your private wealth, and these gentlemen who have too hastily relieved you have only begun in a rude way what must soon be accomplished on a large scale for the benefit of the whole community.' Whereupon we are to suppose the sufferer was much enlightened and refreshed and became a useful member of society. But Christ tells us that it was a Samaritan, a man of property, riding on his own beast and carrying a little capital in his pocket, who lifted up the stranger, bound his wounds, brought him into a place of safety and paid for his support. And He says to every one of us: 'Go thou and do likewise.' Which reading of the parable do you prefer? Which do you follow? It is a serious question. For of two things you may be sure: first, if God has given you possessions in this world they are your own; second, He will certainly hold you to account for what you do with them."—Rev. H. J. Van Dike, Jr. in *Philadelphia Monthly Register.*

